# The presence of Epstein-Barr virus significantly impacts the transcriptional profile in immunodeficiency-associated Burkitt lymphoma

**DOI:** 10.3389/fmicb.2015.00556

**Published:** 2015-06-10

**Authors:** Mohsen Navari, Maryam Etebari, Giulia De Falco, Maria R. Ambrosio, Davide Gibellini, Lorenzo Leoncini, Pier Paolo Piccaluga

**Affiliations:** ^1^Hematopathology Section, Department of Experimental, Diagnostic, and Specialty Medicine, S. Orsola-Malpighi Hospital, Bologna University School of MedicineBologna, Italy; ^2^Department of Basic Sciences, Torbat Heydariyeh University of Medical SciencesTorbat Heydariyeh, Iran; ^3^School of Biological and Chemical Sciences, Queen Mary University of LondonLondon, UK; ^4^Department of Medical Biotechnology, University of SienaSiena, Italy; ^5^Microbiology and Virology Unit, Department of Pathology and Diagnostic, University of VeronaVerona, Italy

**Keywords:** Epstein-Barr virus, latency type, Burkitt lymphoma, HIV/human immunodeficiency virus, microRNA, gene expression profiling

## Abstract

Burkitt lymphoma (BL) is an aggressive neoplasm derived from mature, antigen-experienced B-lymphocytes. Three clinical/epidemiological variants have been recognized, named sporadic, endemic and immunodeficiency-associated BL (ID-BL). Although they are listed within a unique entity in the current WHO Classification, recent evidence indicated genetic and transcriptional differences among the three sub-groups. Further, the presence of latently persisting Epstein-Barr virus (EBV) has been associated with specific features in endemic and sporadic cases. In this study, we explored for the first time whether EBV infection could be related with a specific molecular profile in immunodeficiency-associated cases. We studied 30 BL cases, including nine occurring in HIV-positive patients (5 EBV-positive and 4 EBV-negative) by gene and microRNA (miRNA) expression profiling. We found that ID-BL presented with different profiles based on EBV presence. Specifically, 252 genes were differentially expressed, some of them being involved in intracellular signaling and apoptosis regulation. Furthermore, 28 miRNAs including both EBV-encoded (*N* = 18) and cellular (*N* = 10) ones were differentially regulated. Of note, genes previously demonstrated to be targeted by such miRNA were consistently found among differentially expressed genes, indicating the relevant contribution of miRNA to the molecular profile of the examined cases. Grippingly, 17 out of the 252 differentially expressed genes turned out to be potentially targeted by both cellular and EBV-encoded miRNA, suggesting a complex interaction and not excluding a potential synergism. In conclusion, we documented transcriptional differences based on the presence of EBV in ID-BL, and suggested a complex interaction between cellular and viral molecules in the determination of the global molecular profile of the tumor.

## Introduction

Burkitt lymphoma (BL) is an aggressive lymphoid malignancy derived from antigen experienced B-cells, resembling germinal center (GC) cells (Leucci et al., 2008; Onnis et al., 2012). It represents around 1% of all B-cell non Hodgkin lymphomas, being the most common cancer in children in developing countries but a relatively rare condition in Europe, USA, and Japan (Swerdlow et al., 2008; Simbiri et al., 2014; Stefan, 2015). In the WHO classification it is divided into three clinical/epidemiological variants, namely endemic BL (eBL), sporadic BL (sBL) and immunodeficiency-associated (ID-BL) (Leucci et al., 2008; Swerdlow et al., 2008).

The endemic form is the most common variant, typically occurring in children in the peri-equatorial Africa (Hadley et al., 2012; Stefan, 2015). By contrast, the sporadic and the ID-associated forms occur in western countries more often in young to middle aged adults (Bellan et al., 2003, 2005; Satou et al., 2015). In particular, the ID-associated BL is encountered more often in patients with HIV infection/AIDS or, less frequently, in subjects with congenital or iatrogenic immunodeficiency, including post-transplant immunosuppression (Morscio et al., 2013; Morales-Sanchez and Fuentes-Panana, 2014; Navari et al., 2014). In HIV-infected patients, BL appearance can either be the first sign of manifested AIDS, being among the tumors defining the disease, or develop in more advanced stages of the disease (Bellan et al., 2003; Crosswell et al., 2008). As far as the etiopathogenesis is concerned, immunodeficiency *per se* is considered a risk factor for the development of lymphomas; however, this concept is at least partially challenged by the evidence that BL often arises in HIV patients when the number of circulating T-cells is still within the normal range (Leoncini et al., 2008). On the other hand, evidence suggested that a chronic polymicrobial stimulation, a condition typically observed in immunodeficient subjects, may play a crucial role (Lenoir and Bornkamm, 1987; Van Den Bosch, 2004; Piccaluga et al., 2011).

Among the different pathogens, Epstein-Barr virus (EBV), a very common human herpesvirus which is present in several human malignancies, is currently considered a major player in BL pathogenesis, being documented in around 10–20% of sporadic BL, 30% of ID-BL, and 95% of endemic BL (Niller et al., 2004; Bellan et al., 2005; Hummel et al., 2006; Carbone et al., 2008; Piccaluga et al., 2011; Onnis et al., 2012). However, its exact role is still debated, although virus-induced transformation and the inhibition of apoptosis may be considered as major alternative pathogenetic mechanisms (Niller et al., 2003, 2004). Of note, EBV can adopt different gene expression programs in its latent (non-lytic) state, which are defined based on the expression of 9 viral proteins including both Epstein-Barr Nuclear Antigens (EBNAs) and Latent Membrane Proteins (LMPs). In fact, differently from other lymphomas, including Hodgkin lymphoma, post-transplant lymphoproliferative diseases and EBV-associated T-cell malignancies in which the major EBV-encoded latent oncoproteins like LMP-1 and/or LMP-2 are expressed, BL, according to its typical latency type I program, typically presents with EBNA-1 expression only (Thorley-Lawson and Gross, 2004; Brady et al., 2007; Carbone et al., 2008; Piccaluga et al., 2011; Ghigna et al., 2013; Ito et al., 2014; Murata et al., 2014; Navari et al., 2014; Kim et al., 2015; Vockerodt et al., 2015). In this regard, it has been documented that EBV, through EBNA-1, can manipulate gene and microRNA (miRNA, small RNA molecules with post-transcriptional regulatory role) expression profiles in BL with potential pathogenetic implications like genomic instability (for a recent review see Westhoff Smith and Sugden, 2013), as EBNA-1 can act as a transcription factor. In this context, we documented that hsa-miR-127 overexpression can be induced by EBNA-1 and that the concomitant expression of these two molecules can significantly impair the physiology of memory B-cells (Onnis et al., 2012). The contribution of the resulting miRNAs differentially expressed in EBV-positive vs. EBV-negative BL is so far not clarified (Lenze et al., 2011).

An alternative mechanism, as recently proposed by our group and others, provided evidence that EBV-encoded miRNAs, more than 40 of which are encoded by EBV, might play a pathogenetic role, e.g., through interfering with apoptosis, cell proliferation, cellular miRNA machinery, immune response and metastasis (Choy et al., 2008; De Falco et al., 2009; Skalsky et al., 2012; Ambrosio et al., 2014; Navari et al., 2014; Vereide et al., 2014; Kanda et al., 2015; Kim et al., 2015; Shinozaki-Ushiku et al., 2015). In particular, we showed that EBV-positive BL presented with significant overexpression of EBV-encoded miRNAs belonging to the BART family that were likely to contribute to its global molecular profile (Ambrosio et al., 2014; Navari et al., 2014). Furthermore, we observed that BART6-3p modulation had significant effects on the transcriptome of BL cells and provided evidence that it can affect the expression of relevant proteins including IL-6 receptor, PTEN and WT1, thus inhibiting apoptosis and probably escaping immunosurveillance (Ambrosio et al., 2014).

Another mechanism proposed for the participation of EBV in human malignancies underlines the viral interference with the physiological epigenetic status of the cellular genome (Kang et al., 2002; Grafodatskaya et al., 2010; Caliskan et al., 2011; Hansen et al., 2014; Hernando et al., 2014; Niller et al., 2014).

Nevertheless, most of the studies mainly referred to endemic BL or sporadic BL, and provide us with minor information concerning the molecular pathology of ID-BL (Deffenbacher et al., 2010; Piccaluga et al., 2011; Luzzi et al., 2014).

In this study, we explored the gene and miRNA expression profile of ID-BL, aiming to dissect for the first time the possible contribution of EBV.

## Material and methods

### Ethics statement

The study was conducted in Italy according to the principles of the Helsinki declaration after approval of the Local Review Board.

### Case series

Thirty cases of BL, including 8 eBL, 13 sBL and 9 ID-BL cases, corresponding to 17 EBV-positive and 13 EBV-negative cases, were collected from Italian and African institutes (Supplementary Table 1). The diagnosis was made by at least two expert hematopathologists and confirmed as previously described (Swerdlow et al., 2008; Piccaluga et al., 2011; Navari et al., 2014).

### Gene expression profiling (GEP) analysis

The global GEP of the abovementioned 30 BL cases was prepared from formaldehyde-fixed, paraffin-embedded (FFPE) tissues using DASL (cDNA-mediated Annealing, Selection, extension, and Ligation) whole genome assay, an assay specially designed for FFPE tissues which covers 29,377 human transcripts, as described previously (Piccaluga et al., 2013). In brief, after deparaffinization of FFPE sections using a series of xylene and ethanol washes, total RNA was extracted using RecoverAll™ Total Nucleic Acid Isolation Kit (Life Technologies, Monza, Italy) and quantified using NanoDrop spectrophotometer, and was further converted to cDNA using biotinylated oligo(dT) and random nonamer primers. The biotinylated cDNA was then annealed to the DASL Assay Pool (DAP) probe groups and was further processed according to the manufacturer's instructions. At the end, BeadArray Reader or iScan System was used to determine the presence or absence of specific genes.

Gene expression analysis was carried on as previously reported (Piccaluga et al., 2007, 2008, 2011). The expression value of each selected gene was normalized to have a zero mean value and unit standard deviation. The distance between two individual samples was calculated by Pearson correlation with the normalized expression values. Unsupervised clustering was generated using a hierarchical algorithm based on the average-linkage method. To perform the supervised gene expression analysis, we used GeneSpring GX 12 (Agilent, MI, Italy) and TM4/MeV software version 4.9 using Cosine Correlation (Saeed et al., 2003). Differentially expressed genes between different groups were identified using a two-tails Student *t*-test and adjusted Benjamini-Hochberg correction for false discovery rate, applying the following filtering criteria: *p*-value < 0.05, and fold change >2.

### microRNA expression profiling analysis

microRNA expression profiling, performed on the same cases used for GEP except for one EBV-negative ID-BL case, was achieved using Nanostring nCounter® miRNA Expression Assay Kits (Human V1 miRNA), which detects 654 and 80 human and viral miRNAs, respectively (NanoString Technologies, Seattle, WA, USA) (Supplementary Table 1) as described before (Navari et al., 2014). Raw data was normalized using NanoStringNorm package developed in R 2.15 version: first, probe levels quantified by microarrays were adjusted for miRNAs with specific background correction factor, and were further normalized using geometric mean of positive controls and mean of negative controls for background subtraction. Lastly, the dataset was normalized such that the mean of each gene was zero. The data was further analyzed in the terms of unsupervised Hierarchical Clustering Analysis (HCA) using GeneSpring version GX 12 (Agilent, MI, Italy), as described (Piccaluga et al., 2007, 2008, 2011; Navari et al., 2014). The miRNAs differentially expressed between the two categories were selected on the basis of the following criteria: fold change ≥ 2, corrected *p*-value (Benjamini-Hockeberg FDR) ≤ 0.05. A supervised HCA was then performed as described above.

The gene and miRNA expression profiles of the BL cases were generated using Illumina DASL and Nanostring microarrays, respectively (one experiment for GEP and one for MiRNA). Biological replicates were represented by the different samples (see Supplementary Table 1 for details on each subgroup).

### microRNA target determination

The experimentally validated targets for the viral and cellular miRNAs were searched in public databases. In case of the viral miRNAs, we used VIRmiRNA (http://crdd.osdd.net/servers/virmirna/), a recently established database that contains experimentally validated targets for a wide range of viral miRNAs, including those encoded by EBV (Qureshi et al., 2014). For the cellular miRNAs, we used miRWalk 2 (http://zmf.umm.uni-heidelberg.de/apps/zmf/mirwalk2/), a very recent version of the popular database for miRNA-related research which comprehends several related databases, to collect the experimentally validated targets (Dweep et al., 2011, 2014).

### Gene set enrichment analysis (GSEA) and statistical analysis of the overlapping genes

Gene Set Enrichment Analysis (GSEA) of the interested gene sets was performed in the terms of Gene Ontology (GO) Biological Processes, Oncogenic Signatures and Kyoto Encyclopedia of Genes and Genomes (KEGG) pathways using GSEA MsigDB (www.broadinstitute.org/gsea/msigdb) web-based analysis tool (Mootha et al., 2003; Subramanian et al., 2005), setting the options to the default (displaying top 10 gene sets with FDR *q*-value below 0.05).

Enrichment in expression of EBNA-1 upregulated target genes (Dresang et al., 2009) and EBER-1/2 target genes (Gregorovic et al., 2011) was evaluated using Gene set enrichment analysis (GSEA) software (Mootha et al., 2003; Subramanian et al., 2005) on the set ID-BL samples (EBV-positive vs. EBV-negative, Supplementary Tables 2–3).

The overlapped (shared) genes of the desired sets were extracted and analyzed by GeneSpring GX 12 (Agilent, MI, Italy) for their statistical significance.

## Results

### EBV-positive and EBV-negative ID-BL exhibit different global gene expression profiles

**Figure 1:**
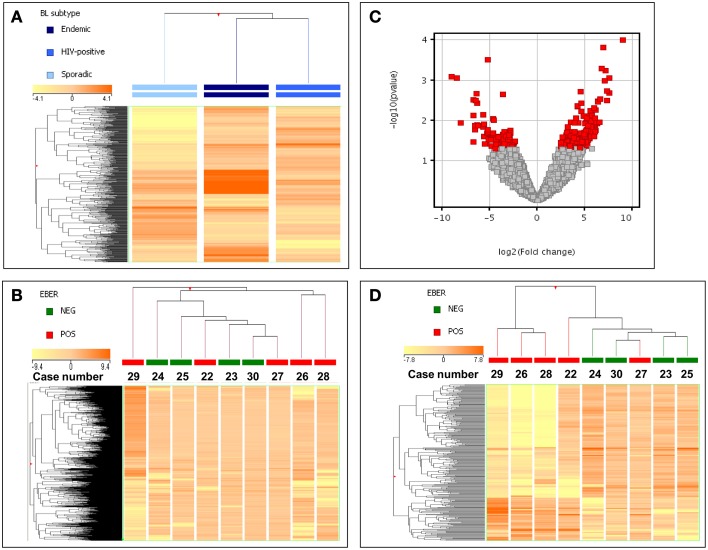
**Gene expression profiling of EBV-pos and EBV-neg ID-BL cases. (A)** An ANOVA analysis demonstrated that BL subtypes are slightly different, with ID-BL being closer to eBL. In the matrix, each column represents a group (mean values recorded in each of the three groups being plotted), and each row represents a gene. The color scale bar shows the relative gene expression changes normalized to the standard deviation (0 is the mean expression level of a given gene). **(B)** Unsupervised hierarchical clustering failed to clearly separate EBV-positive and EBV-negative ID-BL specimens. In the matrix, each column represents a sample, and each row represents a gene. The color scale bar shows the relative gene expression changes normalized to the standard deviation (0 is the mean expression level of a given gene). **(C)** Supervised HCA allowed identifying 252 genes differentially expressed in EBV-positive vs. EBV-negative ID-BL cases (*T*-test, *p* ≤ 0.05; fold change ≥ 2). In the Volcano plot genes with a *p*-value ≤ 0.05 (y-axis, log scale) and fold change ≥2 (x-axis, log scale) are depicted as red squares. **(D)** Based on the expression of the 252 differentiating genes, ID-BL samples were clearly discriminated by a hierarchical clustering according to the EBV presence. In the matrix, each column represents a sample, and each row represents a gene. The color scale bar shows the relative gene expression changes normalized to the standard deviation (0 is the mean expression level of a given gene).

In order to evaluate the possible similarity/differences across ID-BL cases (i.e., EBV-positive vs. EBV-negative), we first looked at the global position of this subtype between the other two subtypes of BL. We analyzed the global gene expression profiles of 30 BL cases, which included 17 EBV-positive and 13 EBV-negative cases (Figure 1A, Supplementary Table 1). A supervised analysis benefiting from the ANOVA (Analysis of Variance, a statistical method for comparing values among three or more groups) method showed that the three categories are relatively similar but distinct, with ID-BL being more similar to the eBL subtype, rather than sBL (Figure 1A). We then proceeded focusing on the ID-BL subtype, as the main goal of our research, and compared the two categories based on the presence/absence of EBV using an unsupervised HCA, which was inefficient in distinguishing between the two categories (Figure 1B).

Aiming at revealing the molecular differences between the two groups of ID-BL, a *t*-Test analysis followed by a fold change filtering was done. Out of 29,377 genes included in the array, we found 69 genes to be downregulated in EBV-negative ID-BL, and 183 genes to be suppressed in EBV-positive ID-BL (Figure 1C, Supplementary Table 4). When these genes were used as the input for a supervised HCA, an efficient discrimination across the two groups was observed (Figure 1D).

**Figure 2:**
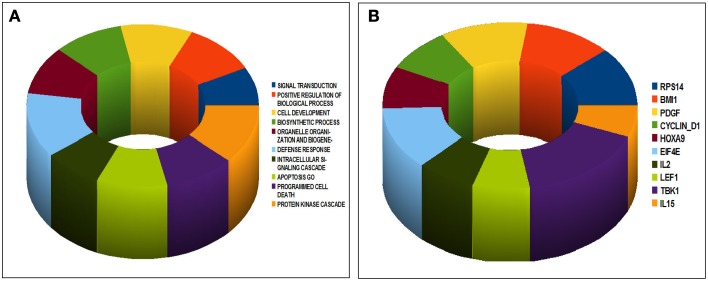
**Gene Set Enrichment Analysis of the differentially expressed genes in ID-BL samples according to the presence of EBV**. The set of the differentiating genes significantly enriched in well recognized Gene Ontology Biological Processes **(A)** or Oncogenic Signatures related to known oncogenes **(B)**.

We then asked whether the genes differentiating our ID-BL cases might have a significant role in tumorigenesis; thus we analyzed them using GSEA MSigDB for GeneOntology (GO) Biological Processes and Oncogenic Signatures (Figures 2A,B). Interestingly, we found enrichment for processes like apoptosis, defense response, protein kinase cascade, programmed cell death and cell development (Figure 2A). Similarly, tumor-related signatures, like Cyclin D1, HOXA9, BMI1 and PDGF were enriched for the deregulated genes (Figure 2B).

### EBV might affect gene and miRNA expression profiles in ID-BL

**Figure 3:**
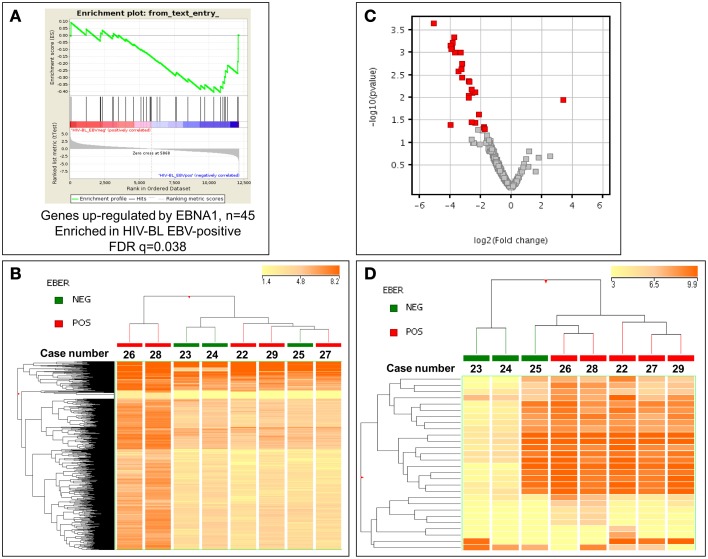
**EBV might interfere with gene and microRNA expression profiling of EBV-positive and EBV-negative ID-BL. (A)** GSEA shows differential expression of the genes up-regulated by EBNA-1 between ID-BL EBV-positive and ID-BL EBV-negative (*n* = 45). GSEA was done with a list of genes up-regulated by EBNA-1 obtained by Dresang et al. (2009). After filtering and normalization, the ~12,000 genes included in the microarray are ranked on the *X axis* (“rank in ordered data set”) of the graph from left to right. Genes on the *left* are up-regulated in EBV-negative cases, whereas the genes ranked to the *right* are up-regulated in EBV-positive cases. *Green line* represents the enrichment score (ES, *Y axis*) with a maximum (absolute value) ES at ~ -0.4; *black vertical lines*, position of individual genes (up-regulated by EBNA-1) from the used gene set in the ordered list of ~ 12,000 genes. To derive significance, the data set was permuted 1000 times and random ES were calculated. The *FDR-q-*value was = 0.038, indicating that the observed distribution is unlikely due to chance. **(B)** Unsupervised analysis failed to discriminate EBV-positive and EBV-negative cases. In the matrix, each column represents a sample, and each row represents a miRNA. The color scale bar shows the relative miRNA expression changes normalized to the standard deviation (0 is the mean expression level of a given miRNA). **(C)**. Supervised analysis identified 28 miRNA differentially expressed between the two groups, with almost all of them to be up-regulated in EBV-positive ID-BL. In the Volcano plot miRNA with a *p*-value ≤ 0.05 (y-axis, log scale) and fold change ≥ 2 (x-axis, log scale) are depicted as red squares. **(D)** Based on the expression of the identified miRNAs, the two groups were clearly discriminated in a HCA. In the matrix, each column represents a sample, and each row represents a miRNA. The color scale bar shows the relative miRNA expression changes normalized to the standard deviation (0 is the mean expression level of a given miRNA).

Since the only known difference in our two groups of tumors is presence/absence of EBV, we then looked for evaluating possible changes that might occur due to the presence of the virus. First, the possible effect of EBNA-1, the only viral protein consistently expressed in BL, was sought using GSEA software, comparing the expression of its targets in EBV-positive and EBV-negative ID-BLs (Figure 3A). Interestingly, a significant enrichment of those targets was observed in EBV-positive ID-BL cases (Figure 3A). Inspired by these results, we further profiled viral and cellular miRNA expression in all BL cases used for GEP, except for one EBV-negative case (Supplementary Table 1). The obtained results were further analyzed in an unsupervised HCA, which demonstrated a relatively similar global miRNA expression profile across the two sets (Figure 3B). However, when a discriminating supervised test was performed, we found 18 EBV-encoded miRNAs (out of 40 viral miRNAs included in the array), all of which belong to BART family of EBV-encoded miRNAs, and nine cellular miRNAs (out of 654 human miRNAs included in the array) to be overexpressed in EBV-positive ID-BL, while, on the other hand, only one cellular miRNA was found to be overexpressed in EBV-negative ID-BL (Table 1, Figure 3C). Of note, these miRNAs could categorize the two sets of tumor samples in a supervised HCA (Figure 3D).

**Table 1:** **microRNAs differentially expressed between EBV-positive and EBV-negative immunodeficiency-related Burkitt Lymphoma**.

**microRNA**	***p*-Value**	**Regulation in EBV-negative ID-BL**	**FC (abs)**	**FC**	**Log FC**	**Accession**
hsa-miR-16	0.039689098	Down	15.61871	−15.618711	−3.9652035	nmiR00181.1
hsa-miR-26a	0.03562978	Down	5.11845	−5.1184497	−2.355707	nmiR00263.1
hsa-miR-142-5p	0.011306038	Up	10.52236	10.522355	3.3953857	nmiR00153.1
hsa-miR-148a	0.045191273	Down	3.461709	−3.4617088	−1.7914844	nmiR00166.1
hsa-miR-200b	0.0489749	Down	3.620994	−3.6209939	−1.8563857	nmiR00227.2
hsa-miR-223	0.03486547	Down	6.085912	−6.085912	−2.6054735	nmiR00257.1
hsa-miR-668	0.046277743	Down	3.499872	−3.4998715	−1.8073019	nmiR00624.1
hsa-miR-877	0.007698981	Down	5.764233	−5.764233	−2.5271287	nmiR00649.1
hsa-miR-1178	0.0076339	Down	5.158424	−5.1584244	−2.3669305	nmiR00020.1
hsa-miR-1233	0.006532014	Down	6.064163	−6.0641627	−2.6003084	nmiR00049.1
ebv-miR-BART1-3p	9.84E-04	Down	12.96857	−12.968566	−3.696947	nmiR00719.1
ebv-miR-BART3	7.82E-04	Down	14.73585	−14.735851	−3.8812585	nmiR00742.1
ebv-miR-BART4	0.00264483	Down	10.94573	−10.945732	−3.4522965	nmiR00743.1
ebv-miR-BART6-3p	0.003621548	Down	9.292624	−9.2926235	−3.216086	nmiR00745.1
ebv-miR-BART6-5p	0.004313744	Down	6.969126	−6.9691257	−2.8009777	nmiR00746.1
ebv-miR-BART7	0.001733595	Down	9.257492	−9.257492	−3.2106214	nmiR00747.1
ebv-miR-BART8	5.99E-04	Down	14.31189	−14.311886	−3.8391418	nmiR00748.1
ebv-miR-BART9	7.05E-04	Down	15.58953	−15.589533	−3.9625058	nmiR00749.1
ebv-miR-BART10	0.008975662	Down	6.915864	−6.9158635	−2.7899094	nmiR00721.1
ebv-miR-BART11-5p	4.62E-04	Down	13.35172	−13.351724	−3.738954	nmiR00723.1
ebv-miR-BART12	0.001006074	Down	10.04338	−10.043382	−3.3281732	nmiR00724.1
ebv-miR-BART15	0.049200244	Down	3.334332	−3.3343318	−1.7373977	nmiR00727.1
ebv-miR-BART17-3p	8.22E-04	Down	15.04786	−15.047857	−3.9114861	nmiR00729.1
ebv-miR-BART17-5p	0.004375282	Down	6.616519	−6.6165185	−2.7260723	nmiR00730.1
ebv-miR-BART18-5p	0.023275565	Down	4.343551	−4.343551	−2.118875	nmiR00732.1
ebv-miR-BART19-3p	0.010132639	Down	6.937569	−6.937569	−2.7944303	nmiR00733.1
ebv-miR-BART21-3p	0.002361224	Down	9.619139	−9.619139	−3.2659078	nmiR00739.1
ebv-miR-BART22	2.28E-04	Down	34.03384	−34.033844	−5.088898	nmiR00741.1

### The deregulated viral and cellular miRNAs might affect the global gene expression profile of EBV-positive ID-BL

**Figure 4:**
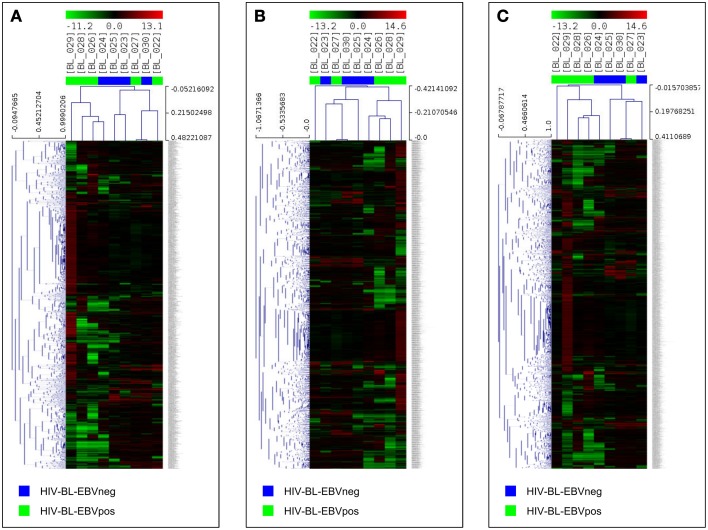
**microRNA targets expression analysis in ID-BL primary cases**. A hierarchical clustering of BL cases based on the expression of genes proved to be targeted by cellular miRNAs **(A**) or EBV-encoded miRNAs (**B**) could roughly discriminate the two subsets of ID-BL. The combination of targets of both cellular and viral miRNAs appeared to be the most effective in clustering the two groups **(C)**. In the matrix, each column represents a sample, and each row represents a gene. The color scale bar shows the relative gene expression changes normalized to the standard deviation (0 is the mean expression level of a given gene).

Since only one cellular miRNA was deregulated in EBV-negative ID-BL, we then focused on the role of the upregulated miRNAs in the other category, i.e., EBV-positive ID-BL. In this regard, experimentally validated targets of those miRNAs were extracted from VIRmiRNA (for EBV-encoded miRNAs) and miRWalk 2 (for cellular miRNAs). A total number of 7654 and 2003 unique targets were found for the differentially expressed cellular and viral miRNAs, corresponding to 4954 and 1293 probes in DASL array, respectively. The effect of the resulting genes on the global gene expression profile of EBV-positive ID-BL was further assessed in a supervised HCA (Figures 4A–C). The results revealed that the targets of viral and cellular miRNAs could roughly distinguish the two groups of ID-BLs (Figures 4A,B, respectively). When these target genes were considered altogether, however, the clustering precision improved, misplacing one sample in each category only (Figure 4C).

**Figure 5:**
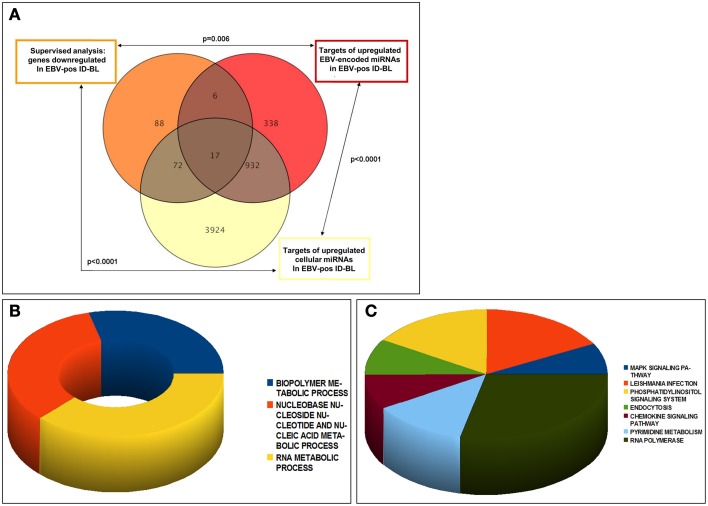
**Analysis of the experimentally validated targets of differentially expressed cellular and viral miRNAs**. Genes targeted by the differentially expressed miRNAs between EBV-positive and EBV-negative ID-BLs were significantly over-represented among genes differentially expressed between the same two tumor categories. Of note, several targets were in common between cellular and viral miRNAs (**A**). The genes targeted by either cellular or viral miRNAs and down-regulated in EBV-positive ID-BL (*n* = 95) were further analyzed for Gene Ontology Biological Process (**B**) and KEGG pathways (**C**).

These target genes, related to either upregulated cellular or viral miRNAs, were in addition investigated for recognizing the genes common between them, or among them and genes downregulated in EBV-positive ID-BL, and the overlap significance was calculated statistically. The results are presented in Figure 5A. Of note, we found 89 genes of the potential targets of the deregulated cellular miRNAs to be underexpressed in EBV-positive ID-BL (*p* < 0.0001, Supplementary Table 5). In addition, 23 of downregulated genes in EBV-positive ID-BL were observed among targets of BART miRNAs (*p* = 0.006, Supplementary Table 6). Interestingly, we found 949 common genes between the targets of human and EBV-encoded miRNAs (*p* < 0.0001), 17 of which were downregulated in EBV-positive ID-BL (Supplementary Table 7).

To estimate the role of the deregulated miRNAs in EBV-positive ID-Burkitt lymphomagenesis, the genes targeted by either human or EBV miRNAs were assessed for GO Biological Processes and KEGG pathways. Regarding the processes, we saw enrichment for metabolic-related processes like biopolymers, nucleic acids and RNA molecules (Figure 5B). Concerning KEGG pathways, on the other hand, the noted results included MAPK, phosphatidylinositol and chemokine signaling pathways (Figure 5C).

## Discussion

Since discovery of EBV numerous studies have been performed to elucidate the role of this virus in different human malignancies, especially in associated lymphomas (for a recent review see Vockerodt et al., 2015). Although such a role looks to be more definable in the context of tumors like Post-Transplant Lymphoproliferative Disorder (PTLD) or Hodgkin's Lymphoma, where the virus expresses its oncoproteins like LMP-1, the contribution of EBV to BL is still debated (Niller et al., 2003, 2004; Hummel et al., 2006; Carbone et al., 2008; Morscio et al., 2013; Morales-Sanchez and Fuentes-Panana, 2014; Navari et al., 2014). However, several mechanisms like genomic instability induced by EBNA-1 or inhibition of apoptosis, induction of metastasis and cell growth, interfering with cellular miRNA machinery and escaping immunosurveillance directed by miRNAs targeting proteins like PUMA, BIM, BAD, PTEN, and IL6ST among others have been proposed (Choy et al., 2008; Gruhne et al., 2009; Marquitz et al., 2011; Skalsky et al., 2012; Ambrosio et al., 2014; Kanda et al., 2015; Kim et al., 2015; Shinozaki-Ushiku et al., 2015). While infection of EBV alongside with HIV, a phenomenon observed in 30% of ID-BL cases, adds another dimension to this uncertainty, at the same time it represents an excellent opportunity for unraveling the contribution of EBV to ID-BL and more generally to other tumors associated with HIV AND EBV, e.g., a subset of PTLDs (Shimoyama et al., 2006; Carbone et al., 2008; Morovic et al., 2009; Morscio et al., 2013).

Here we used a combination of experimental data and bioinformatic tools to investigate the differences between EBV-positive and EBV-negative ID-BL cases. In this regards, we evaluated BL samples in the terms of both gene and miRNA expression profiling. We found that BL subtypes are similar, but distinct, and that EBV-positive and EBV-negative ID-BL samples differ in their gene and miRNA expression profiles. Furthermore, we showed that EBV, through EBNA-1 and its miRNAs, might be able to manipulate the global molecular profile of EBV-positive ID-BLs, and that the deregulated viral and cellular miRNAs in this tumor might co-target different genes.

In line with a previous study performed by our laboratory we found that global gene expression profiles of ID-BLs are more similar to eBL, rather than sBL (Lenze et al., 2011; Piccaluga et al., 2011). As discussed before, this similarity might be based on the similar predisposition of the patients to infectious agents, i.e., Malaria and HIV, for eBL and ID-BL, respectively (Van Den Bosch, 2004; Piccaluga et al., 2011). Of note, in the abovementioned project we used fresh frozen samples for defining such profiles, while here we used FFPE tissues. These results, besides the nature of the outcome, served us also as a control for our experiment, since FFPE tissues normally result in low-quality RNA not appropriate for most microarray platforms, and indicate once more the appropriateness of DASL technology for FFPE tissues (Piccaluga et al., 2013; Laginestra et al., 2014a).

Our incapability to discriminate between EBV-positive and EBV-negative ID-BLs in an unsupervised analysis was expected somehow, as the two groups belong to the same category (Leucci et al., 2010; Piccaluga et al., 2011). Consistently, we found a moderate number of genes to be differentially expressed between the two groups, which could differentiate the two sets of tumors very well. Although little in number, however, the differentiating genes turned out to be enriched in tumor-related biological processes and signaling pathways; a fact that could be interpreted as different tumorigenic mechanisms in the two sides leading to the same pathological phenotype, a phenomenon already described in human tumors, e.g., activated B-cell (ABC) and germinal center B (GCB) phenotypes of diffuse large B-cell lymphoma (Blenk et al., 2007; Dasmahapatra et al., 2012).

Concerned with the role of EBV, we showed that EBNA-1, as the only EBV-encoded latent protein of EBV consistently present in BL, is able to modulate the global gene expression profile of EBV-positive ID-BLs. It is known that EBNA-1, the prime role of which is maintaining the EBV episome inside the nucleus, binds to cellular DNA and can alter the transcriptional pattern of the host cells, affecting both genes and miRNAs (Wood et al., 2007; Dresang et al., 2009; Onnis et al., 2012). It has also been suggested that EBNA-1 might induce genomic instability caused by reactive oxygen species (ROS) (Gruhne et al., 2009). Furthermore, in murine models EBV has shown B-cell lymphoma inducing properties (Wilson et al., 1996).

By contrast, quite surprisingly, when we sought for a possible enrichment in EBERs (1 and 2) targets (Gregorovic et al., 2011) within the genes characteristic of EBV-positive ID-BL, we did not find any significant enrichment. This might be due to the fact that we referred to EBER targets as identified in lymphoblastoid cells (Gregorovic et al., 2011). This model, for any reason, might be suboptimal for our system. Alternatively, it might be that in BL molecular programs similar to the ones induced/repressed by EBERs are anyway affected by genomic imbalances, including *MYC* abnormalities themselves, this making EBV-positive and EBV-negative cases rather similar in this respect. Further, the presence of HIV and the possible associated immune hyper stimulation might affect the cellular programs mimicking the EBER effects, again inducing similar molecular phenotypes in EBV-positive and EBV-negative cases.

EBV encodes more than 40 miRNAs and several reports highlight the role of those miRNAs in human tumorigenesis, like evading the immune system, resistance to apoptosis and induction of metastasis (Ramalingam et al., 2012; Cullen, 2013; Ambrosio et al., 2014; Andrade et al., 2014; Qiu and Thorley-Lawson, 2014; Kanda et al., 2015; Qiu et al., 2015; Shinozaki-Ushiku et al., 2015). In addition, we have previously demonstrated how those miRNAs, especially one named ebv-miR-BART6-3p, could influence the global gene expression profile of EBV-positive BLs (Navari et al., 2014). Thus, we proceeded with investigation of miRNA profiling in our samples and found several cellular and viral miRNAs to discriminate EBV-positive and EBV-negative ID-BLs. We did not find any miRNAs from the BHRF family, which was expectable, as they seem to be expressed in latency type III of EBV (Xia et al., 2008; Qiu et al., 2011; Navari et al., 2014). Instead, several miRNAs belonging to the BART family (both cluster 1 and cluster 2) were found to be upregulated in EBV-positive ID-BL. Interestingly, all these miRNAs except three of them (namely ebv-miR-BART15, 18-5p and 19-5p) were in common with the set we described comparing EBV-positive BL and EBV-positive PTLD-DLBCL (Navari et al., 2014). This confirms our previous findings regarding low expression or absence of expression of these miRNAs in EBV-positive PTLD-DLBCL, which displays latency type III of EBV (Navari et al., 2014).

When the cases were clustered using the discriminating miRNAs, curiously we found that one of the EBV-negative cases (BL_25) was clustered together with EBV-positive ones. Although unexpected, this might be related to the presence of an EBER-deleted genome, or more generally to lack of the sensitivity of EBER *in situ* hybridization in this specific case. However, it should be also noted that by GEP it clustered within EBV-negative cases as it did another EBV-positive case (BL_22). This might reflect the presence of genetic lesions, additional to *MYC* translocation, that seem to occur more often in EBV-negative cases than in EBV-positive ones (Laginestra et al., 2014b).

A previous study by Lenze et al. (2011) compared the cellular miRNAs expression levels between EBV-positive and EBV-negative BL cases, irrespective of the BL subtypes, and not including the viral miRNAs, as we did in the present study. Interestingly, they reported no miRNA discriminating the two subgroups of BL. Similarly, we found only one cellular miRNA differentiating the same two categories (Piccaluga et al., submitted). By contrast, when in the present study we focused on ID-BL cases (i.e., studied the effect of EBV in the presence of HIV on the miRNA profile—not done by Lenze et al.), we found 10 differentially expressed cellular miRNAs.

These results inspired us to ask if the presence of HIV, when combined with the presence of EBV, might influence the expression level of cellular miRNAs.

Although normally HIV does not infect B-cells, 95% of all lymphomas described in HIV-infected individuals are of B-cell origin (Swerdlow et al., 2008). A change in the host microenvironment induced by HIV infection and abnormalities in B-cells of infected individuals has been reported (Cheng et al., 2009). It has been speculated that such changes in the microenvironment are mainly exerted by HIV-encoded products, which are released from the infected cells and taken up by the uninfected cells. This has been well proved at least for a soluble form of HIV-Tat protein, for which transforming properties have been suggested (Frankel and Pabo, 1988; Chen et al., 1995; Ma and Nath, 1997). Indeed, we have found positivity for HIV-Tat in B-cell lymphomas, as proved by immunohistochemistry (Lazzi et al., 2002). Furthermore, our recent findings indicate an active role for this protein in disrupting cellular gene and miRNA expression in ID-BL, primarily exerted through DNA methyl transferase enzymes (Luzzi et al., 2014). Based on these findings we speculate that co-expression of EBV- and HIV-encoded proteins (and possibly miRNAs) in ID-BL might explain the high number of induced cellular miRNAs, when compared to EBV-negative ID-BL cases.

As miRNAs, both viral and human, seemed to be major players in EBV-positive ID-BL, we further investigated their experimentally validated targets and their effect on transcriptional profile of EBV-positive ID-BL. The results of a supervised HCA indicated that the differentiating ability of the targets of these miRNAs, i.e., cellular or viral, improved when both of them were included in the analysis. These outcomes could indicate that the transcriptional profile of the tumor could be deregulated by both miRNA groups, instead of single sets. When common genes between different gene sets, including targets of viral and human miRNAs and genes downregulated in EBV-positive ID-BL were searched, they significantly overlapped. The overlapping set of genes between targets of EBV or human miRNAs and genes downregulated in the EBV-positive tumor cases turned out to be enriched in metabolic processes, comparable to our previous results (Navari et al., 2014), and cancer-related pathways, an indication of the possible importance of these targets in lymphomagenesis of EBV + ID-BL.

The interaction between cellular and viral miRNAs could be defined in a complex network of interactions which might contain both synergism and antagonism among the miRNAs (Lutter et al., 2010; Xu et al., 2011). Although viral miRNAs generally share low homology with human miRNAs, some do not follow the general rule and thus are considered as orthologs to human miRNAs (Babu et al., 2011). For example, miR-K12-11, a miRNA encoded by Kaposi's-sarcoma-associated herpes virus (KSHV) is a well-proven ortholog of the human oncogenic miRNA has-miR-155 (Gottwein et al., 2007; Skalsky et al., 2007; Boss et al., 2011), and ebv-miR-BART-5 might be an ortholog of hsa-miR-18 and act as a viral oncogenic miRNA in human cells (Babu et al., 2011). In addition, it has been suggested that some of these viral miRNAs, despite low global homology, might have shared target sites with human ones, probably due to homology in their seed sequence (Babu et al., 2011; Andrade et al., 2014). For instance, ebv-miR-BART15 is reported target *NLRP3* at the same binding site of has-miR-223, conferring to the virus the ability to regulate the inflammation (Haneklaus et al., 2012). Furthermore, common targets for human and EBV miRNAs with different binding sites have been reported (Riley et al., 2012). Very interestingly, in our results we found a very high number of genes which would be targeted by both human and viral miRNAs, 17 of which were suppressed in EBV-positive ID-BL. Of note, the possible contribution of lowered expression of several of these genes in human tumors is already proven, like PAFAH1B1 in non-small cell lung cancer, SMARCC1 in pancreatic cancer and NKN2 in myeloproliferative neoplasm (MPN) precursors (Lo et al., 2012; Iwagami et al., 2013; Mehrotra et al., 2013). These findings might indicate a synergism between the viral and induced human miRNAs, although more experimental data are needed in support of such a hypothesis.

From an evolutionary point of view, the two viruses, although not infecting the same cell type, could be considered competitors, as they both manipulate their microenvironment through release of their encoded products from infected cells (like miRNAs in case of EBV and Tat in case of HIV) which might not necessarily be favorable for each other. However, on the other hand, a synergy or favorable pathogenetic mechanism between the two viruses might resolve such a conflict and help the viruses to improve their chance of survival. Such a phenomenon has been long known for plant viruses (Pruss et al., 1997), and has been suggested between HIV and herpes simplex virus 1 (Heng et al., 1994; Cheng and Nixon, 2009) and HIV and Hepatitis-B Virus (Sun et al., 2014).

Based on our findings, we hypothesize that the outcome of the co-infection with both viruses might be different from the sum of them in singularity, as it is a matter of virus-virus and virus- host complex interaction networks. However, this hypothesis needs further support by experimental models.

In conclusion we showed for the first time that the presence of EBV significantly affects the transcriptional profile of ID-BL, in terms of both genes and miRNAs. Particularly, EBV appeared to exert its influence through both EBNA-1 and viral miRNAs. Finally, differently from sporadic and endemic BL, cellular miRNAs turned out to be largely deregulated in the presence of EBV. Future studies should address the specific relationship between genes and miRNAs, as well as the potential interaction among HIV-encoded, EBV-encoded, and cellular miRNAs and proteins.

## Financial support

This work was supported by the Centro Interdipartimentale per la Ricerca sul Cancro “G. Prodi”, BolognAIL, AIRC IG 2013 N.14355—Prof. Piccaluga, RFO (Prof. Piccaluga), Progetto Strategico di Ateneo 2006 (Prof. Piccaluga), and FIRB Futura 2011 RBFR12D1CB (Prof. Piccaluga).

### Conflict of interest statement

The authors declare that the research was conducted in the absence of any commercial or financial relationships that could be construed as a potential conflict of interest.

## References

[B1] Ambrosio M. R. Navari M. Di Lisio L. Leon E. A. Onnis A. Gazaneo S.. (2014). The Epstein Barr-encoded BART-6-3p microRNA affects regulation of cell growth and immuno response in Burkitt lymphoma. Infect Agent Cancer 9, 12. 10.1186/1750-9378-9-12 24731550 PMC4005456

[B2] Andrade T. A. Evangelista A. F. Campos A. H. Poles W. A. Borges N. M. Camillo C. M.. (2014). A microRNA signature profile in EBV+ diffuse large B-cell lymphoma of the elderly. Oncotarget 5, 11813–11826. 25544772 10.18632/oncotarget.2952 PMC4322989

[B3] Babu S. G. Ponia S. S. Kumar D. Saxena S. (2011). Cellular oncomiR orthologue in EBV oncogenesis. Comput. Biol. Med. 41, 891–898. 10.1016/j.compbiomed.2011.07.007 21880309

[B4] Bellan C. De Falco G. Lazzi S. Leoncini L. (2003). Pathologic aspects of AIDS malignancies. Oncogene 22, 6639–6645. 10.1038/sj.onc.1206815 14528289

[B5] Bellan C. Lazzi S. Hummel M. Palummo N. De Santi M. Amato T.. (2005). Immunoglobulin gene analysis reveals 2 distinct cells of origin for EBV-positive and EBV-negative Burkitt lymphomas. Blood 106, 1031–1036. 10.1182/blood-2005-01-0168 15840698

[B6] Blenk S. Engelmann J. Weniger M. Schultz J. Dittrich M. Rosenwald A.. (2007). Germinal center B cell-like (GCB) and activated B cell-like (ABC) type of diffuse large B cell lymphoma (DLBCL): analysis of molecular predictors, signatures, cell cycle state and patient survival. Cancer Inform. 3, 399–420. 19455257 PMC2675856

[B7] Boss I. W. Nadeau P. E. Abbott J. R. Yang Y. Mergia A. Renne R. (2011). A Kaposi's sarcoma-associated herpesvirus-encoded ortholog of microRNA miR-155 induces human splenic B-cell expansion in NOD/LtSz-scid IL2Rgammanull mice. J. Virol. 85, 9877–9886. 10.1128/JVI.05558-11 21813606 PMC3196388

[B8] Brady G. Macarthur G. J. Farrell P. J. (2007). Epstein-Barr virus and Burkitt lymphoma. J. Clin. Pathol. 60, 1397–1402. 10.1136/jcp.2007.047977. 18042696 PMC2095571

[B9] Caliskan M. Cusanovich D. A. Ober C. Gilad Y. (2011). The effects of EBV transformation on gene expression levels and methylation profiles. Hum. Mol. Genet. 20, 1643–1652. 10.1093/hmg/ddr041 21289059 PMC3063990

[B10] Carbone A. Gloghini A. Dotti G. (2008). EBV-associated lymphoproliferative disorders: classification and treatment. Oncologist 13, 577–585. 10.1634/theoncologist.2008-0036 18515742

[B11] Chen L. L. Frankel A. D. Harder J. L. Fawell S. Barsoum J. Pepinsky B. (1995). Increased cellular uptake of the human immunodeficiency virus-1 Tat protein after modification with biotin. Anal. Biochem. 227, 168–175. 10.1006/abio.1995.1267 7668378

[B12] Cheng R. G. Nixon D. F. (2009). Herpes simplex virus and HIV-1: deciphering viral synergy. Lancet Infect. Dis. 9, 74–74. 10.1016/S1473-3099(09)70002-7 19179220

[B13] Cheng S. M. Li J. C. Lin S. S. Lee D. C. Liu L. Chen Z.. (2009). HIV-1 transactivator protein induction of suppressor of cytokine signaling-2 contributes to dysregulation of IFN{gamma} signaling. Blood 113, 5192–5201. 10.1182/blood-2008-10-183525 19279332

[B14] Choy E. Y. Siu K. L. Kok K. H. Lung R. W. Tsang C. M. To K. F.. (2008). An Epstein-Barr virus-encoded microRNA targets PUMA to promote host cell survival. J. Exp. Med. 205, 2551–2560. 10.1084/jem.20072581 18838543 PMC2571930

[B15] Crosswell H. E. Bergsagel D. J. Yost R. Lew G. (2008). Successful treatment with modified CHOP-rituximab in pediatric AIDS-related advanced stage Burkitt lymphoma. Pediatr. Blood Cancer 50, 883–885. 10.1002/pbc.21161 17278123

[B16] Cullen B. R. (2013). MicroRNAs as mediators of viral evasion of the immune system. Nat. Immunol. 14, 205–210. 10.1038/ni.2537 23416678 PMC3642974

[B17] Dasmahapatra G. Lembersky D. Son M. P. Patel H. Peterson D. Attkisson E.. (2012). Obatoclax interacts synergistically with the irreversible proteasome inhibitor carfilzomib in GC- and ABC-DLBCL cells *in vitro* and *in vivo*. Mol. Cancer Ther. 11, 1122–1132. 10.1158/1535-7163.MCT-12-0021 22411899 PMC3601737

[B18] De Falco G. Antonicelli G. Onnis A. Lazzi S. Bellan C. Leoncini L. (2009). Role of EBV in microRNA dysregulation in Burkitt lymphoma. Semin. Cancer Biol. 19, 401–406. 10.1016/j.semcancer.2009.07.003 19619656

[B19] Deffenbacher K. E. Iqbal J. Liu Z. Fu K. Chan W. C. (2010). Recurrent chromosomal alterations in molecularly classified AIDS-related lymphomas: an integrated analysis of DNA copy number and gene expression. J. Acquir. Immune Defic. Syndr. 54, 18–26. 10.1097/qai.0b013e3181d3d9eb 20216076

[B20] Dresang L. R. Vereide D. T. Sugden B. (2009). Identifying sites bound by Epstein-Barr virus nuclear antigen 1 (EBNA1) in the human genome: defining a position-weighted matrix to predict sites bound by EBNA1 in viral genomes. J. Virol. 83, 2930–2940. 10.1128/JVI.01974-08 19129441 PMC2655553

[B21] Dweep H. Gretz N. Sticht C. (2014). miRWalk database for miRNA-target interactions. Methods Mol. Biol. 1182, 289–305. 10.1007/978-1-4939-1062-5_25 25055920

[B22] Dweep H. Sticht C. Pandey P. Gretz N. (2011). miRWalk–database: prediction of possible miRNA binding sites by “walking” the genes of three genomes. J. Biomed. Inform. 44, 839–847. 10.1016/j.jbi.2011.05.002 21605702

[B23] Frankel A. D. Pabo C. O. (1988). Cellular uptake of the tat protein from human immunodeficiency virus. Cell 55, 1189–1193. 10.1016/0092-8674(88)90263-2 2849510

[B24] Ghigna M. R. Reineke T. Rince P. Schuffler P. El Mchichi B. Fabre M.. (2013). Epstein-Barr virus infection and altered control of apoptotic pathways in posttransplant lymphoproliferative disorders. Pathobiology 80, 53–59. 10.1159/000339722 22868923

[B25] Gottwein E. Mukherjee N. Sachse C. Frenzel C. Majoros W. H. Chi J.-T. A.. (2007). A viral microRNA functions as an orthologue of cellular miR-155. Nature 450, 1096–1099. 10.1038/nature05992 18075594 PMC2614920

[B26] Grafodatskaya D. Choufani S. Ferreira J. C. Butcher D. T. Lou Y. Zhao C.. (2010). EBV transformation and cell culturing destabilizes DNA methylation in human lymphoblastoid cell lines. Genomics 95, 73–83. 10.1016/j.ygeno.2009.12.001 20005943

[B27] Gregorovic G. Bosshard R. Karstegl C. E. White R. E. Pattle S. Chiang A. K.. (2011). Cellular gene expression that correlates with EBER expression in Epstein-Barr Virus-infected lymphoblastoid cell lines. J. Virol. 85, 3535–3545. 10.1128/JVI.02086-10 21248031 PMC3067860

[B28] Gruhne B. Sompallae R. Marescotti D. Kamranvar S. A. Gastaldello S. Masucci M. G. (2009). The Epstein-Barr virus nuclear antigen-1 promotes genomic instability via induction of reactive oxygen species. Proc. Natl. Acad. Sci. U.S.A. 106, 2313–2318. 10.1073/pnas.0810619106 19139406 PMC2650153

[B29] Hadley L. G. Rouma B. S. Saad-Eldin Y. (2012). Challenge of pediatric oncology in Africa. Semin. Pediatr. Surg. 21, 136–141. 10.1053/j.sempedsurg.2012.01.006 22475119

[B30] Haneklaus M. Gerlic M. Kurowska-Stolarska M. Rainey A. A. Pich D. Mcinnes I. B.. (2012). Cutting edge: miR-223 and EBV miR-BART15 regulate the NLRP3 inflammasome and IL-1beta production. J. Immunol. 189, 3795–3799. 10.4049/jimmunol.1200312 22984081

[B31] Hansen K. D. Sabunciyan S. Langmead B. Nagy N. Curley R. Klein G.. (2014). Large-scale hypomethylated blocks associated with Epstein-Barr virus-induced B-cell immortalization. Genome Res. 24, 177–184. 10.1101/gr.157743.113 24068705 PMC3912409

[B32] Heng M. C. Heng S. Y. Allen S. G. (1994). Co-infection and synergy of human immunodeficiency virus-1 and herpes simplex virus-1. Lancet 343, 255–258. 10.1016/S0140-6736(94)91110-X 7905094

[B33] Hernando H. Islam A. B. Rodriguez-Ubreva J. Forne I. Ciudad L. Imhof A.. (2014). Epstein-Barr virus-mediated transformation of B cells induces global chromatin changes independent to the acquisition of proliferation. Nucleic Acids Res. 42, 249–263. 10.1093/nar/gkt886 24097438 PMC3874198

[B34] Hummel M. Bentink S. Berger H. Klapper W. Wessendorf S. Barth T. F.. (2006). A biologic definition of Burkitt's lymphoma from transcriptional and genomic profiling. N. Engl. J. Med. 354, 2419–2430. 10.1056/NEJMoa055351 16760442

[B35] Ito T. Kawazu H. Murata T. Iwata S. Arakawa S. Sato Y.. (2014). Role of latent membrane protein 1 in chronic active Epstein-Barr virus infection-derived T/NK-cell proliferation. Cancer Med. 3, 787–795. 10.1002/cam4.256 24799376 PMC4303147

[B36] Iwagami Y. Eguchi H. Nagano H. Akita H. Hama N. Wada H.. (2013). miR-320c regulates gemcitabine-resistance in pancreatic cancer via SMARCC1. Br. J. Cancer 109, 502–511. 10.1038/bjc.2013.320 23799850 PMC3721395

[B37] Kanda T. Miyata M. Kano M. Kondo S. Yoshizaki T. Iizasa H. (2015). Clustered microRNAs of the Epstein-Barr virus cooperatively downregulate an epithelial cell-specific metastasis suppressor. J. Virol. 89, 2684–2697. 10.1128/JVI.03189-14 25520514 PMC4325718

[B38] Kang G. H. Lee S. Kim W. H. Lee H. W. Kim J. C. Rhyu M. G.. (2002). Epstein-barr virus-positive gastric carcinoma demonstrates frequent aberrant methylation of multiple genes and constitutes CpG island methylator phenotype-positive gastric carcinoma. Am. J. Pathol. 160, 787–794. 10.1016/S0002-9440(10)64901-2 11891177 PMC1867170

[B39] Kim H. Choi H. Lee S. K. (2015). Epstein-Barr virus miR-BART20-5p regulates cell proliferation and apoptosis by targeting BAD. Cancer Lett. 356, 733–742. 10.1016/j.canlet.2014.10.023 25449437

[B40] Laginestra M. A. Abate F. Etebari M. De Falco G. Fuligni F. Rossi M. (2014b). Identification of single-nucleotide variants by high-throughput RNA sequencing in endemic Burkitt Lymphoma, in Proceedings of the 105th Annual Meeting of the American Association for Cancer Research (San Diego; Philadelphia).

[B41] Laginestra M. A. Piccaluga P. P. Fuligni F. Rossi M. Agostinelli C. Righi S.. (2014a). Pathogenetic and diagnostic significance of microRNA deregulation in peripheral T-cell lymphoma not otherwise specified. Blood Cancer J. 4, 259. 10.1038/bcj.2014.78 25382608 PMC4335255

[B42] Lazzi S. Bellan C. De Falco G. Cinti C. Ferrari F. Nyongo A.. (2002). Expression of RB2/p130 tumor-suppressor gene in AIDS-related non-Hodgkin's lymphomas: implications for disease pathogenesis. Hum. Pathol. 33, 723–731. 10.1053/hupa.2002.125372 12196924

[B43] Lenoir G. M. Bornkamm G. W. (1987). Burkitt's Lymphoma, a human cancer model for the study of the multistep development of cancer: proposal for a new scenario, in Advances in Viral Oncology, ed Klein G. (New York, NY: Raven Press), 173–206.

[B44] Lenze D. Leoncini L. Hummel M. Volinia S. Liu C. G. Amato T.. (2011). The different epidemiologic subtypes of Burkitt lymphoma share a homogenous micro RNA profile distinct from diffuse large B-cell lymphoma. Leukemia 25, 1869–1876. 10.1038/leu.2011.156 21701491 PMC3902789

[B45] Leoncini L. Raphaël M. Stein H. Harris N. L. Jaffe E. S. Kluin P. M. (2008). Burkitt lymphoma, in WHO Classification of Tumors of the Hematopoietic and Lymphoid Tissue, eds Swerdlow S. Campo E. Harris N. L. Jaffe E. S. Pileri S. A. Stein H. Thiele J. Vardiman J. (Lyon: IARC), 262–264.

[B46] Leucci E. Cocco M. Onnis A. De Falco G. Van Cleef P. Bellan C.. (2008). MYC translocation-negative classical Burkitt lymphoma cases: an alternative pathogenetic mechanism involving miRNA deregulation. J. Pathol. 216, 440–450. 10.1002/path.2410 18802929

[B47] Leucci E. Onnis A. Cocco M. De Falco G. Imperatore F. Giuseppina A.. (2010). B-cell differentiation in EBV-positive Burkitt lymphoma is impaired at posttranscriptional level by miRNA-altered expression. Int. J. Cancer 126, 1316–1326. 10.1002/ijc.24655 19530237

[B48] Lo F. Y. Chen H. T. Cheng H. C. Hsu H. S. Wang Y. C. (2012). Overexpression of PAFAH1B1 is associated with tumor metastasis and poor survival in non-small cell lung cancer. Lung Cancer 77, 585–592. 10.1016/j.lungcan.2012.05.105 22749159

[B49] Lutter D. Marr C. Krumsiek J. Lang E. W. Theis F. J. (2010). Intronic microRNAs support their host genes by mediating synergistic and antagonistic regulatory effects. BMC Genomics 11:224. 10.1186/1471-2164-11-224 20370903 PMC2865499

[B50] Luzzi A. Morettini F. Gazaneo S. Mundo L. Onnis A. Mannucci S.. (2014). HIV-1 Tat induces DNMT over-expression through microRNA dysregulation in HIV-related non Hodgkin lymphomas. Infect. Agents Cancer 9, 41. 10.1186/1750-9378-9-41 25705251 PMC4334912

[B51] Ma M. Nath A. (1997). Molecular determinants for cellular uptake of Tat protein of human immunodeficiency virus type 1 in brain cells. J. Virol. 71, 2495–2499. 9032389 10.1128/jvi.71.3.2495-2499.1997 PMC191362

[B52] Marquitz A. R. Mathur A. Nam C. S. Raab-Traub N. (2011). The Epstein-Barr Virus BART microRNAs target the pro-apoptotic protein Bim. Virology 412, 392–400. 10.1016/j.virol.2011.01.028 21333317 PMC3340891

[B53] Mehrotra S. Sharma B. Joshi S. Kroczynska B. Majchrzak B. Stein B. L.. (2013). Essential role for the Mnk pathway in the inhibitory effects of type I interferons on myeloproliferative neoplasm (MPN) precursors. J. Biol. Chem. 288, 23814–23822. 10.1074/jbc.M113.476192 23814052 PMC3745328

[B54] Mootha V. K. Lepage P. Miller K. Bunkenborg J. Reich M. Hjerrild M. (2003). Identification of a gene causing human cytochrome c oxidase deficiency by integrative genomics. Proc. Natl. Acad. Sci. USA. 100, 605–610. 10.1073/pnas.242716699 12529507 PMC141043

[B55] Morales-Sanchez A. Fuentes-Panana E. M. (2014). Human viruses and cancer. Viruses 6, 4047–4079. 10.3390/v6104047 25341666 PMC4213577

[B56] Morovic A. Jaffe E. S. Raffeld M. Schrager J. A. (2009). Metachronous EBV-associated B-cell and T-cell posttransplant lymphoproliferative disorders in a heart transplant recipient. Am. J. Surg. Pathol. 33, 149–154. 10.1097/PAS.0b013e318181a826 18941401 PMC6324846

[B57] Morscio J. Dierickx D. Ferreiro J. F. Herreman A. Van Loo P. Bittoun E.. (2013). Gene expression profiling reveals clear differences between EBV-positive and EBV-negative posttransplant lymphoproliferative disorders. Am. J. Transplant 13, 1305–1316. 10.1111/ajt.12196 23489474

[B58] Murata T. Sato Y. Kimura H. (2014). Modes of infection and oncogenesis by the Epstein-Barr virus. Rev. Med. Virol. 24, 242–253. 10.1002/rmv.1786 24578255

[B59] Navari M. Fuligni F. Laginestra M. A. Etebari M. Ambrosio M. R. Sapienza M. R.. (2014). Molecular signature of Epstein Barr virus-positive Burkitt lymphoma and post-transplant lymphoproliferative disorder suggest different roles for Epstein Barr virus. Front. Microbiol. 5:728. 10.3389/fmicb.2014.00728 25566237 PMC4274971

[B60] Niller H. H. Salamon D. Ilg K. Koroknai A. Banati F. Bauml G.. (2003). The *in vivo* binding site for oncoprotein c-Myc in the promoter for Epstein-Barr virus (EBV) encoding RNA (EBER) 1 suggests a specific role for EBV in lymphomagenesis. Med. Sci. Monit. 9, HY1-9. 12552250

[B61] Niller H. H. Salamon D. Ilg K. Koroknai A. Banati F. Schwarzmann F.. (2004). EBV-associated neoplasms: alternative pathogenetic pathways. Med. Hypotheses 62, 387–391. 10.1016/j.mehy.2003.11.001 14975509

[B62] Niller H. H. Szenthe K. Minarovits J. (2014). Epstein-Barr virus-host cell interactions: an epigenetic dialog? Front. Genet. 5:367. 10.3389/fgene.2014.00367 25400657 PMC4212275

[B63] Onnis A. Navari M. Antonicelli G. Morettini F. Mannucci S. De Falco G.. (2012). Epstein-Barr nuclear antigen 1 induces expression of the cellular microRNA hsa-miR-127 and impairing B-cell differentiation in EBV-infected memory B cells. New insights into the pathogenesis of Burkitt lymphoma. Blood Cancer J. 2, e84. 10.1038/bcj.2012.29 22941339 PMC3432484

[B64] Piccaluga P. P. Agostinelli C. Califano A. Carbone A. Fantoni L. Ferrari S.. (2007). Gene expression analysis of angioimmunoblastic lymphoma indicates derivation from T follicular helper cells and vascular endothelial growth factor deregulation. Cancer Res. 67, 10703–10710. 10.1158/0008-5472.CAN-07-1708 18006812

[B65] Piccaluga P. P. Califano A. Klein U. Agostinelli C. Bellosillo B. Gimeno E.. (2008). Gene expression analysis provides a potential rationale for revising the histological grading of follicular lymphomas. Haematologica 93, 1033–1038. 10.3324/haematol.12754 18492688

[B66] Piccaluga P. P. De Falco G. Kustagi M. Gazzola A. Agostinelli C. Tripodo C.. (2011). Gene expression analysis uncovers similarity and differences among Burkitt lymphoma subtypes. Blood 117, 3596–3608. 10.1182/blood-2010-08-301556 21245480

[B67] Piccaluga P. P. Fuligni F. De Leo A. Bertuzzi C. Rossi M. Bacci F.. (2013). Molecular profiling improves classification and prognostication of nodal peripheral T-cell lymphomas: results of a phase III diagnostic accuracy study. J. Clin. Oncol. 31, 3019–3025. 10.1200/JCO.2012.42.5611 23857971

[B68] Pruss G. Ge X. Shi X. M. Carrington J. C. Vance V. B. (1997). Plant viral synergism: the potyviral genome encodes a broad-range pathogenicity enhancer that transactivates replication of heterologous viruses. Plant Cell 9, 859–868. 10.1105/tpc.9.6.859 9212462 PMC156963

[B69] Qiu J. Cosmopoulos K. Pegtel M. Hopmans E. Murray P. Middeldorp J.. (2011). A novel persistence associated EBV miRNA expression profile is disrupted in neoplasia. PLoS Pathog. 7:e1002193. 10.1371/journal.ppat.1002193 21901094 PMC3161978

[B70] Qiu J. Smith P. Leahy L. Thorley-Lawson D. A. (2015). The Epstein-Barr virus encoded BART miRNAs potentiate tumor growth *in vivo*. PLoS Pathog. 11:e1004561. 10.1371/journal.ppat.1004561 25590614 PMC4295875

[B71] Qiu J. Thorley-Lawson D. A. (2014). EBV microRNA BART 18-5p targets MAP3K2 to facilitate persistence *in vivo* by inhibiting viral replication in B cells. Proc. Natl. Acad. Sci. U.S.A. 111, 11157–11162. 10.1073/pnas.1406136111 25012295 PMC4121837

[B72] Qureshi A. Thakur N. Monga I. Thakur A. Kumar M. (2014). VIRmiRNA: a comprehensive resource for experimentally validated viral miRNAs and their targets. Database (Oxford) 2014, 1–10. 10.1093/database/bau103 25380780 PMC4224276

[B73] Ramalingam D. Kieffer-Kwon P. Ziegelbauer J. M. (2012). Emerging themes from EBV and KSHV microRNA targets. Viruses 4, 1687–1710. 10.3390/v4091687 23170179 PMC3499826

[B74] Riley K. J. Rabinowitz G. S. Yario T. A. Luna J. M. Darnell R. B. Steitz J. A. (2012). EBV and human microRNAs co-target oncogenic and apoptotic viral and human genes during latency. EMBO J. 31, 2207–2221. 10.1038/emboj.2012.63 22473208 PMC3343464

[B75] Saeed A. I. Sharov V. White J. Li J. Liang W. Bhagabati N.. (2003). TM4: a free, open-source system for microarray data management and analysis. Biotechniques 34, 374–378. 12613259 10.2144/03342mt01

[B76] Satou A. Asano N. Nakazawa A. Osumi T. Tsurusawa M. Ishiguro A.. (2015). Epstein-Barr virus (EBV)-positive sporadic burkitt lymphoma: an age-related lymphoproliferative disorder? Am. J. Surg. Pathol. 39, 227–235. 10.1097/PAS.0000000000000332 25321330

[B77] Shimoyama Y. Nakamura S. Asano N. Oshiro A. Oyama T. (2006). [Epstein-Barr virus (EBV)-associated lymphomas and lymphoproliferative disorders]. Nippon. Rinsho 64(Suppl. 3), 635–638. 16615550

[B78] Shinozaki-Ushiku A. Kunita A. Isogai M. Hibiya T. Ushiku T. Takada K.. (2015). Profiling of virus-encoded MicroRNAs in Epstein-Barr virus-associated gastric carcinoma and their roles in gastric carcinogenesis. J. Virol. 89, 5581–5591. 10.1128/JVI.03639-14 25740983 PMC4442544

[B79] Simbiri K. O. Biddle J. Kinyera T. Were P. A. Tenge C. Kawira E.. (2014). Burkitt lymphoma research in East Africa: highlights from the 9(th) African organization for research and training in cancer conference held in Durban, South Africa in 2013. Infect. Agents Cancer 9, 32. 10.1186/1750-9378-9-32 25686906 PMC4163050

[B80] Skalsky R. L. Corcoran D. L. Gottwein E. Frank C. L. Kang D. Hafner M.. (2012). The viral and cellular microRNA targetome in lymphoblastoid cell lines. PLoS Pathog. 8:e1002484. 10.1371/journal.ppat.1002484 22291592 PMC3266933

[B81] Skalsky R. L. Samols M. A. Plaisance K. B. Boss I. W. Riva A. Lopez M. C.. (2007). Kaposi's sarcoma-associated herpesvirus encodes an ortholog of miR-155. J. Virol. 81, 12836–12845. 10.1128/JVI.01804-07 17881434 PMC2169101

[B82] Stefan D. C. (2015). Patterns of distribution of childhood cancer in Africa. J. Trop. Pediatr. [Epub ahead of print]. 10.1093/tropej/fmv005 25724211

[B83] Subramanian A. Tamayo P. Mootha V. K. Mukherjee S. Ebert B. L. Gillette M. A.. (2005). Gene set enrichment analysis: a knowledge-based approach for interpreting genome-wide expression profiles. Proc. Natl. Acad. Sci. U.S.A. 102, 15545–15550. 10.1073/pnas.0506580102 16199517 PMC1239896

[B84] Sun H. Y. Sheng W. H. Tsai M. S. Lee K. Y. Chang S. Y. Hung C. C. (2014). Hepatitis B virus coinfection in human immunodeficiency virus-infected patients: a review. World J. Gastroenterol. 20, 14598–14614. 10.3748/wjg.v20.i40.14598 25356024 PMC4209527

[B85] Swerdlow S. H. Campo E. Harris N. L. Jaffe E. S. Pileri S. A. Stein H. (2008). WHO Classification of Tumours of Haematopoietic and Lymphoid Tissues, Fourth Edn. Lyon: IARC Press

[B86] Thorley-Lawson D. A. Gross A. (2004). Persistence of the Epstein-Barr virus and the origins of associated lymphomas. N. Engl. J. Med. 350, 1328–1337. 10.1056/NEJMra032015 15044644

[B87] Van Den Bosch C. A. (2004). Is endemic Burkitt's lymphoma an alliance between three infections and a tumour promoter? Lancet Oncol. 5, 738–746. 10.1016/S1470-2045(04)01650-X 15581545

[B88] Vereide D. T. Seto E. Chiu Y. F. Hayes M. Tagawa T. Grundhoff A.. (2014). Epstein-Barr virus maintains lymphomas via its miRNAs. Oncogene 33, 1258–1264. 10.1038/onc.2013.71 23503461 PMC3690170

[B89] Vockerodt M. Yap L. F. Shannon-Lowe C. Curley H. Wei W. Vrzalikova K.. (2015). The Epstein-Barr virus and the pathogenesis of lymphoma. J. Pathol. 235, 312–322. 10.1002/path.4459 25294567

[B90] Westhoff Smith D. Sugden B. (2013). Potential cellular functions of Epstein-Barr Nuclear Antigen 1 (EBNA1) of Epstein-Barr Virus. Viruses 5, 226–240. 10.3390/v5010226 23325328 PMC3564119

[B91] Wilson J. B. Bell J. L. Levine A. J. (1996). Expression of Epstein-Barr virus nuclear antigen-1 induces B cell neoplasia in transgenic mice. EMBO J. 15, 3117–3126. 8670812 PMC450254

[B92] Wood V. H. O'neil J. D. Wei W. Stewart S. E. Dawson C. W. Young L. S. (2007). Epstein-Barr virus-encoded EBNA1 regulates cellular gene transcription and modulates the STAT1 and TGFbeta signaling pathways. Oncogene 26, 4135–4147. 10.1038/sj.onc.1210496 17486072

[B93] Xia T. O'hara A. Araujo I. Barreto J. Carvalho E. Sapucaia J. B.. (2008). EBV microRNAs in primary lymphomas and targeting of CXCL-11 by ebv-mir-BHRF1-3. Cancer Res. 68, 1436–1442. 10.1158/0008-5472.CAN-07-5126 18316607 PMC2855641

[B94] Xu J. Li C. X. Li Y. S. Lv J. Y. Ma Y. Shao T. T.. (2011). MiRNA-miRNA synergistic network: construction via co-regulating functional modules and disease miRNA topological features. Nucleic Acids Res. 39, 825–836. 10.1093/nar/gkq832 20929877 PMC3035454

